# Drug-target interaction prediction based on spatial consistency constraint and graph convolutional autoencoder

**DOI:** 10.1186/s12859-023-05275-3

**Published:** 2023-04-17

**Authors:** Peng Chen, Haoran Zheng

**Affiliations:** 1 School of Computer Science and Technology, University of Science and Technology of China, Jinzhai Road 96, Hefei, 230027 People’s Republic of China; 2 Anhui Key Laboratory of Software Engineering in Computing and Communication, University of Science and Technology of China, Jinzhai Road 96, Hefei, 230027 People’s Republic of China

**Keywords:** Drug-target interaction, Spatial consistency constraint, Graph convolutional autoencoder, Deep learning

## Abstract

**Background:**

Drug-target interaction (DTI) prediction plays an important role in drug discovery and repositioning. However, most of the computational methods used for identifying relevant DTIs do not consider the invariance of the nearest neighbour relationships between drugs or targets. In other words, they do not take into account the invariance of the topological relationships between nodes during representation learning. It may limit the performance of the DTI prediction methods.

**Results:**

Here, we propose a novel graph convolutional autoencoder-based model, named SDGAE, to predict DTIs. As the graph convolutional network cannot handle isolated nodes in a network, a pre-processing step was applied to reduce the number of isolated nodes in the heterogeneous network and facilitate effective exploitation of the graph convolutional network. By maintaining the graph structure during representation learning, the nearest neighbour relationships between nodes in the embedding space remained as close as possible to the original space.

**Conclusions:**

Overall, we demonstrated that SDGAE can automatically learn more informative and robust feature vectors of drugs and targets, thus exhibiting significantly improved predictive accuracy for DTIs.

**Supplementary Information:**

The online version contains supplementary material available at 10.1186/s12859-023-05275-3.

## Background

Drug-target interaction (DTI) prediction plays a significant role in drug discovery and repositioning [1, 2]. Many investigations on drug side effects, poly-pharmacology and drug resistance rely on DTI predictions [3]. However, biochemical experiments to identify DTIs can be expensive and time consuming [4]. Alternatively, computational approaches can effectively identify potential clinically valuable DTIs with significantly reduced costs.

Early traditional computational methods can be divided into two categories, one based on molecular docking [5] and the other based on ligands [6]. However, when the 3D structure of the target protein is unknown, the performance of the methods based on molecular docking are limited. In addition, when the target has only a small number of known binding ligands, the methods based on ligands perform poorly. In the past decade, much effort has been devoted to develop machine learning-based methods to predict potential DTIs. Xuan et al. [7] proposed a prediction method based on non-negative matrix factorisation and a gradient boosting tree model, which can make fully utilise negative samples to learn low-dimensional representations of drugs and targets. Ezzat et al. [8] proposed another matrix factorisation-based method named GRMF, which introduces graph regularisation into low-rank approximation to improve the prediction performance of the algorithm. DTINet was proposed by Luo et al. [9] to integrate information from heterogeneous data sources, and thus capture topological information of drugs and targets from various networks to obtain low-dimensional feature vectors.

However, these shallow machine learning methods have limited learning capabilities, which can hamper their ability to capture the relationship between features and DTIs. Deep learning is a type of machine learning that plays a significant role in speech recognition [10] and image processing [11], and is able to deal with complex biomedical and chemical problems [12, 13] owing to its multi-layered and non-linear structures. Therefore, in recent years, DTI prediction based on deep learning has become a research hotspot.

Based on different input features, deep learning-based DTI prediction methods can be broadly divided into three branches: ligand-, structure-, and relationship-based methods [14]. In particular, ligand-based methods leverage the ligand information of the tested target and use deep learning approaches to simplify the virtual screening steps. In turn, structure-based methods use information from both the target proteins and their ligands. For example, the first application of deep learning for DTI prediction was demonstrated by Wen et al. [15], who developed the DeepDTIs. It extracts potential features of drugs and targets based on unsupervised pre-training using raw descriptors. Subsequently, Öztürk et al. [16] proposed DeepDTA, a convolutional neural network-based model that uses Simplified Molecular Input Line Entry System (SMILES) information of drugs and the amino acid sequence of proteins to predict DTIs, which outperformed the previously reported KronRLS [17] and SimBoosts [18] models. More recently, Huang et al. [19] proposed an augmented Transformer [20] encoder-based method for extracting and capturing semantic relations among substructures of drugs and targets from a large amount of unlabelled biological data.

Heterogeneous data sources provide diverse information and multiple perspectives for the prediction of novel DTIs [9]. Relationship-based methods use heterogeneous networks to integrate information from multi-source biological data among drugs, proteins, diseases, side effects and so on. Zhao et al. [21] proposed DLDTI, which is based on network representation learning and convolutional neural networks. It can incorporate interaction information, attribute characteristics, and network topology of each node in a complex network. The model then uses the learned low-dimensional and informative vectors to perform DTI prediction. In turn, Peng et al. [22] used the Jaccard similarity coefficients [23] and random walk with restart (RWR) [24] to extract the drug and target features, along with a denoising autoencoder to select the network-based features and reduce the dimensionality of the feature representation. Notably, many relationship-based prediction models use graph convolutional networks (GCNs). For example, Manoochehri et al. [25] proposed an end-to-end model in which a heterogeneous network with seven types of edges, comprising drugs, proteins, and diseases, was constructed and graph convolution was performed for each edge type. Liu et al [26] also proposed a model, named GADTI, based on a graph convolutional autoencoder. The encoder in this model consists of the combination of a GCN and an RWR, which provides more information to the nodes, and DisMult [27] was used as the decoder. The GANDTI model proposed by Sun et al. [28] also uses a GCN to encode the drug and target features, but it then uses a generative adversarial network (GAN) to enhance the robustness and reduce the noise of feature vectors. However, most of these methods do not maintain invariant neighbour relationships during representation learning. It is possible that the nearest neighbour relationships between nodes are shifted in the embedding space. These changes may negatively affect the prediction results. At the same time, most of these current methods cannot handle nodes that are not present in the network. In fact, there are a large number of unknown drugs and targets represented as isolated nodes in the interaction network. Therefore, how to process the isolated nodes is a challenge that has to be overcome to achieve more accurate DTI predictions.

Herein, we propose SDGAE, a graph convolutional autoencoder-based DTI prediction method that was designed to address the limitations of the current approaches. SDGAE first uses the Weighted *K* Nearest Known Neighbours (WKNKN) algorithm to densify the DTI matrix and reduce the number of isolated nodes in a heterogeneous network. During the encoding process, we added spatial consistency constraint (SCC) to the model, which ensures that the topological relationships between nodes in the embedding space remains as close as possible to the original space. Finally, based on ensemble learning, a LightGBM [29] model was constructed for DTI prediction.

The innovations and contribution of this paper can be concluded as follows:

1. By introducing SCC during representation learning, the original topology of the node is preserved in the embedding space. Therefore, the nearest neighbour relationships between nodes in the embedding space remain as close as possible to the original space.
2. A pre-processing step for densifying DTI matrix is introduced before graph convolution. Isolated nodes in heterogeneous network are fully considered and dealt with, thus further exploiting the effectiveness of GCN.
3. Our work provides a new research idea for the optimisation of DTI prediction methods based on graph neural network encoding.

## Materials

The dataset used in this study was obtained from public databases, as described previously [9], comprising 1923 known DTIs (i.e. positive samples) and 1,068,573 negative samples. The quantity and source of the nodes in the dataset are shown in Table 1. Among the 708 drugs and 1512 targets included in the dataset, 159 drugs and 1088 targets did not have known interactions, called $'$unknown drugs$'$ and $'$unknown targets$'$, respectively. The drugs/targets that had known interactions with at least one target/drug were called $'$known drugs$'$ and $'$known targets$'$, respectively. Hence, a set of drugs $D=\left\{ d_{i} \mid i=1, \ldots , m\right\}$ and targets $T=\left\{ t_{j} \mid j=1, \ldots , n\right\}$ were contained in the dataset, where *m* and *n* represent the number of drugs and targets, respectively. The DTIs are represented by a binary matrix $Y \in R^{m \times n}$. If there was a known interaction between drug $d_{i}$ and target $t_{j}$, then $Y(i, j)=1$.

**Table 1:** Details of the dataset

Type	Quantity	Source
Drug	708	DrugBank (Version 3.0) [30]
Protein	1512	HPRD (Release 9) [31]
Disease	5603	CTD [32]
Side effect	4192	SIDER (Version 2) [33]

## Methods

### Overview of SDGAE

**Fig. 1:**
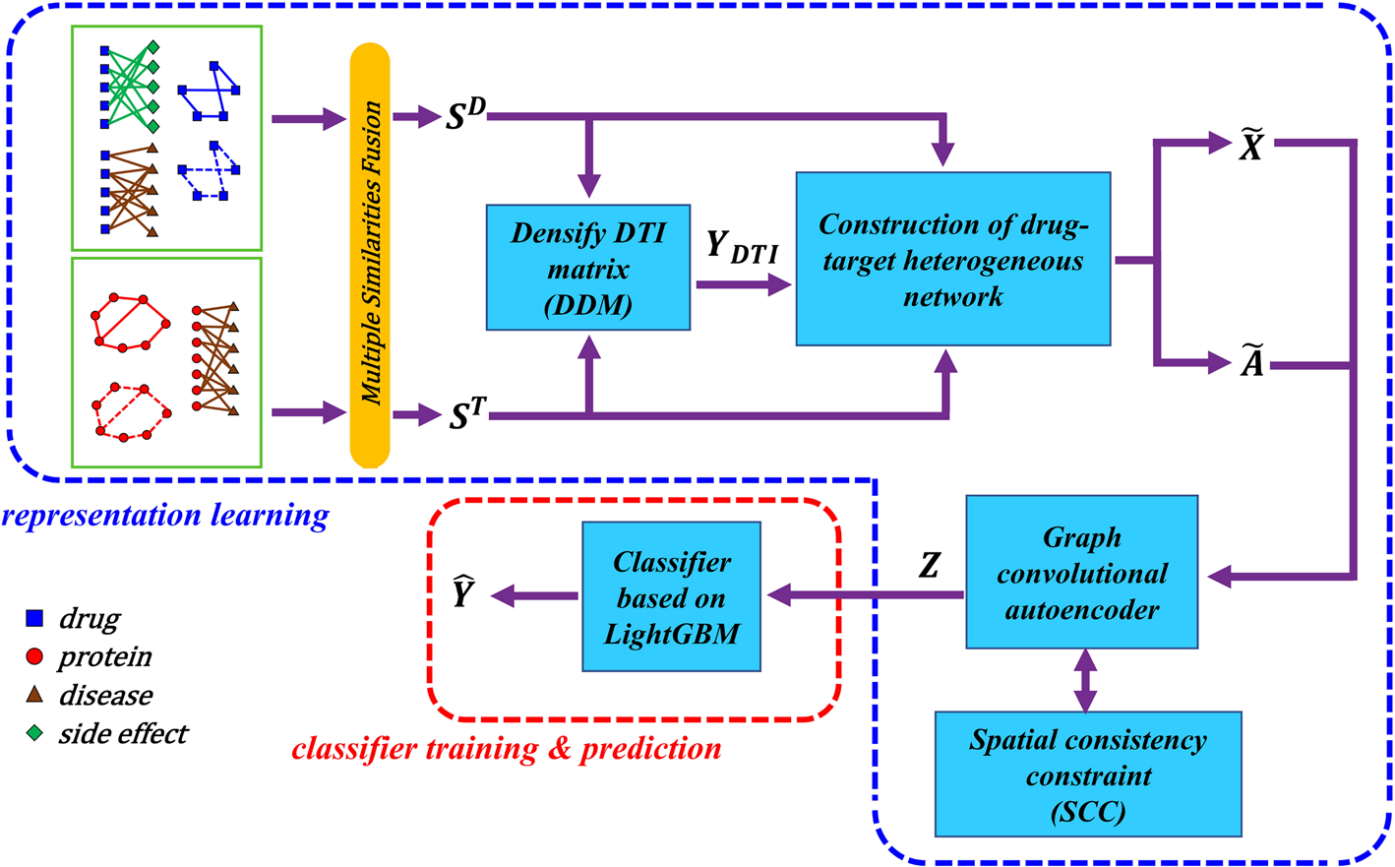
Flowchart of SDGAE

The overall workflow of SDGAE is shown in Fig. 1. SDGAE consisted of two stages: a representation learning stage, and a classifier training & prediction stage. During the representation learning stage, the networks related to drugs or targets were processed through a multiple similarities fusion step to obtain the similarity matrices $S^{D}$ and $S^{T}$, respectively. These two matrices were then used for densifying DTI matrix and construction of drug-target heterogeneous network. Then, SDGAE was designed to generate an adjacency matrix $\widetilde{A}$ and node feature matrix $\widetilde{X}$, which were used for the subsequent graph convolutional autoencoder. In addition, a SCC was introduced in the process of autoencoding. Finally, the graph convolutional autoencoder generated the feature vector matrix *Z* for drugs and targets. During the classifier training & prediction stage, a LightGBM-based classifier was constructed and trained using the feature vector matrix *Z*.

### Multiple similarities fusion

A similarity matrix between drugs (calculated from chemical structures) and targets (calculated from amino acid sequences) already existed in the dataset, denoted as $S_{chemical}^{D} \in R^{m\times m}$ and $S_{sequence}^{T} \in R^{n\times n}$ respectively. Given that the nearest neighbour relationships between nodes in the embedding space needed to be as consistent as possible with those of the original space, the similarity in the original space was considered highly significant. We considered it unilateral to use only one source of data to measure the similarity between nodes. Thus, we measured and fused multiple types of similarity calculated from various sources.

For drug-drug interactions (DDIs), drug-disease associations and drug-side effect associations, we calculated the similarity between two drugs based on the Jaccard similarity coefficient. Considering the drug-side effect association network as an example, the similarity between $d_{i}$ and $d_{j}$ was calculated using the following equation:

1 $$\begin{aligned} \begin{aligned} S_{sideeffect}^{D}(i,j)=\frac{\left| SE_i \cap SE_j\right| }{\left| SE_i \cup S E_j\right| } \end{aligned} \end{aligned}$$

where $SE_i$ represents the set of side effects associated with the drug $d_{i}$. Therefore, the similarity of all drugs concerning side effects was obtained and denoted by the matrix $S_{sideeffect}^{D} \in R^{m\times m}$, in which each element of the matrix represents the similarity between two drugs, with values close to 1 indicating that the two drugs are similar. The same process was performed for the DDI network and the drug-disease association network to obtain the corresponding similarity matrices, denoted as $S_{interaction}^{D}$ and $S_{disease}^{D} \in R^{m\times m}$, respectively.

To measure the similarity between targets from multiple perspectives, the same process was performed for the target-target interaction (TTI) network and target-disease association network to obtain two similarity matrices for targets, which were denoted as $S_{interaction}^{T}$ and $S_{disease}^{T} \in R^{n \times n}$, respectively.

The fusion similarity matrices for the drugs ($S^{D} \in R^{m\times m}$) and targets ($S^{T} \in R^{n\times n}$) were then obtained using Eqs. (2) and (3), respectively.

2 $$\begin{aligned} S^D(i, j)= & {} \max \left( S_{chemical}^{D}(i, j), \ S_{interaction}^{D}(i, j), \ S_{disease}^{D}(i,j), \ S_{sideeffect}^{D}(i,j) \right) \end{aligned}$$

3 $$\begin{aligned} S^T(i, j)= & {} \max \left( S_{sequence}^{T}(i, j), \ S_{interaction}^{T}(i, j), \ S_{disease}^{T}(i,j) \right) \end{aligned}$$

### Densify DTI matrix (DDM)

In the study dataset, only 1923 (0.1796%) drug-target pairs were known to have an interaction. Unknown drugs and targets (See "Materials" Section) behaved as isolated nodes in the DTI network. Because GCN cannot handle isolated nodes based on local neighbourhood information, the existence of these isolated nodes limits the DTI prediction methods based on GCN. If the interactions of these unknown drugs and targets can be inferred according to other drugs or targets before GCN, the number of isolated nodes in the heterogeneous network can be reduced. Thus, the performance of DTI prediction method based on GCN may be greatly improved. Based on the assumption that molecules with similar chemical structures may interact with the same molecules, SDGAE designed the following strategy for densifying DTI matrix.

In the DTI matrix *Y* (See "Materials" Section), the *i*-th row represents the interaction profile of the drug $d_{i}$ and all targets, denoted as $Y(d_i)=\{Y(i, 1), Y(i, 2) \cdots Y(i, n)\}$. In turn, the *j*-th column in *Y* represents the interaction profile of the target $t_{j}$ and all drugs, which is denoted as $Y(t_j)=\{Y(1, j), Y(2, j) \cdots Y(m, j)\}$. Some drug-target pairs are not found to interact (zeros in *Y*) but they potentially interact (i.e. false negative samples). Therefore, the WKNKN algorithm was designed to use known DTIs to estimate the likelihood of unexplored DTIs. After the algorithm, some of the zeros in *Y* were replaced by values between 0 and 1. The larger the value, the more likely was to exist an interaction between the drug and the target. Hence, using WKNKN, we obtained a densified matrix $Y_{dense} \in R^{m \times n}$. Algorithm 1 shows the main steps.
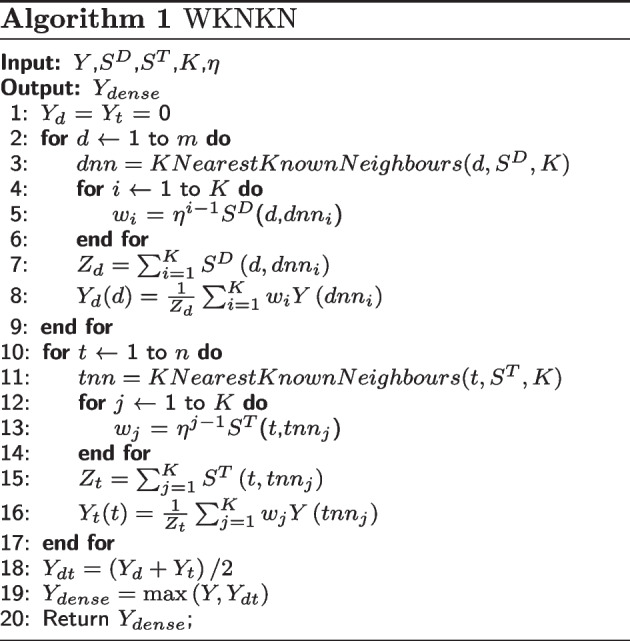


*KNearestKnownNeighbours*() returns the *K*-nearest neighbours of a drug or target in descending order based on the similarity matrix $S^{D}$ or $S^{T}$. Notably, when returning the *K*-nearest neighbours of a drug, only known drugs were considered, whereas unknown drugs were excluded, because the interaction profiles of unknown drugs were all zeros, and they could not provide useful interaction information (the same was true for targets).

After the above-described steps, some zeros in the *Y* matrix were replaced with values between 0 and 1, which are denoted as $E=\{e_1, e_2 \cdots \}$. These values were sorted in ascending order and the median value $e_{median}$ was selected as the threshold value. Thus a discretized DTI matrix $Y_{DTI} \in R^{m \times n}$ was obtained according to the following equation:

4 $$\begin{aligned} \begin{aligned} Y_{DTI}= {\left\{ \begin{array}{ll}0 &{} Y_{dense }<e_{median } \\ 1 &{} Y_{dense } \ge e_{median}\end{array}\right. } \end{aligned} \end{aligned}$$

### Construction of drug-target heterogeneous network

$D=\left\{ d_{i} \mid i=1, \ldots , m\right\}$ was used to represent *m* drug nodes and $T=\left\{ t_{j} \mid j=1, \ldots , n\right\}$ was used to represent *n* target nodes. A DDI network was constructed based on the drug-drug interactions: if two drugs interacted, an edge was connected between the two drug nodes. The DDI network was denoted by an adjacency matrix $A^{D} \in R^{m\times m}$. If there was an interaction between the drug $d_i$ and drug $d_j$, then $A^{D}(i, j)=1$, otherwise $A^{D}(i, j)=0$. Similarly, a TTI network was constructed and represented by $A^{T} \in R^{n\times n}$. To jointly exploit the drug and target interaction information, if the drug $d_i$ and target $t_j$ were identified in $Y_{DTI}$ as interacting (i.e. $Y_{DTI}(i, j)=1$), an edge was added between drug node $d_i$ and target node $t_j$. Thus, a drug-target heterogeneous network was constructed by connecting the DDI and TTI network through the $Y_{DTI}$ matrix.

**Fig. 2:**
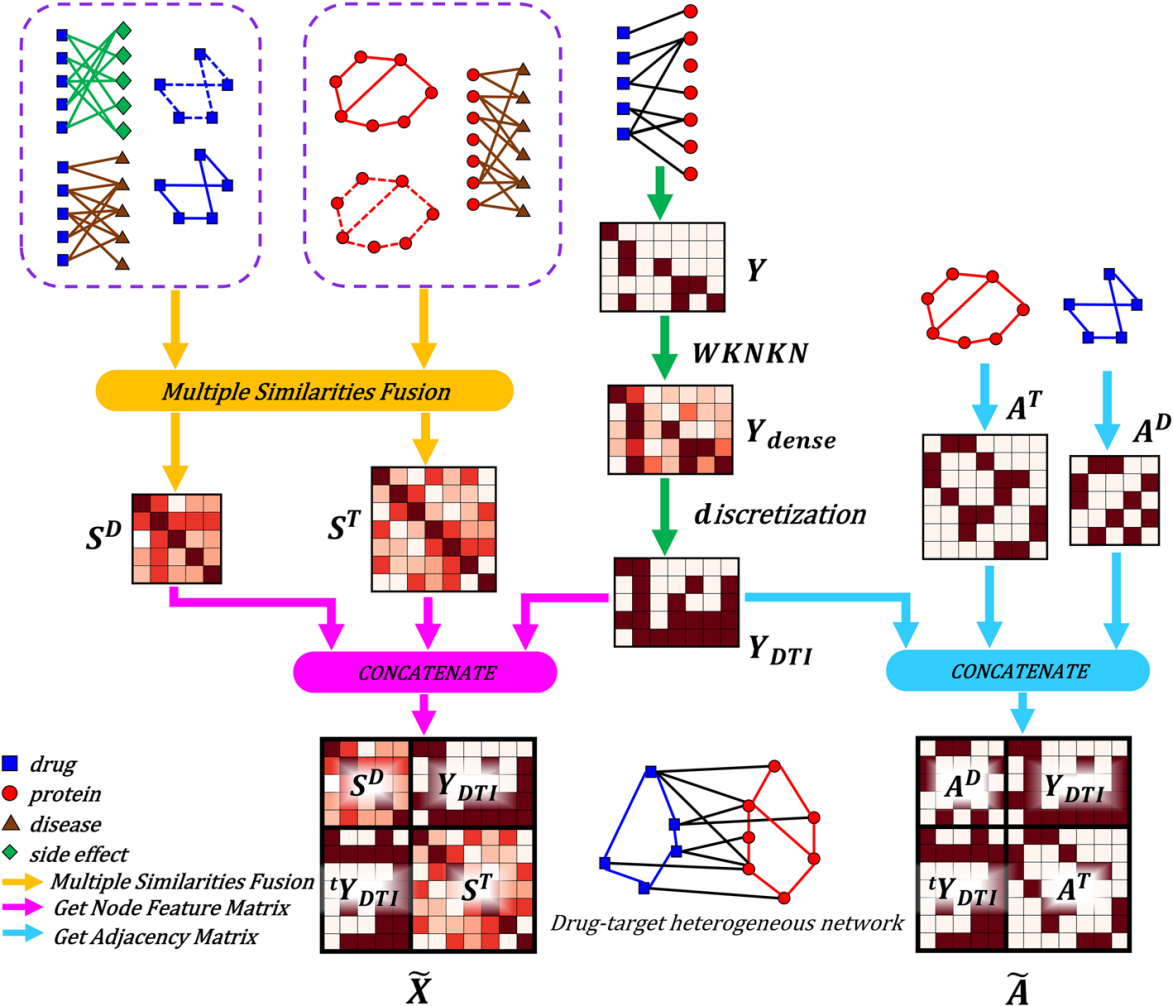
Multiple similarities fusion and construction of heterogeneous network

As $A^{D}$, $A^{T}$, and $Y_{DTI}$ contained the topological information of the heterogeneous network, the topological adjacency matrix $\widetilde{A} \in R^{(m+n) \times (m+n)}$ of the heterogeneous network was obtained by concatenating these three matrices (Fig. 2, where ${ }^t Y_{DTI}$ represents the transpose of $Y_{DTI}$). $\widetilde{A}$ and $\widetilde{X}$ were used as the adjacency matrix and node feature matrix for the subsequent graph convolutional encoder, respectively.

### Graph convolutional encoder

**Fig. 3:**
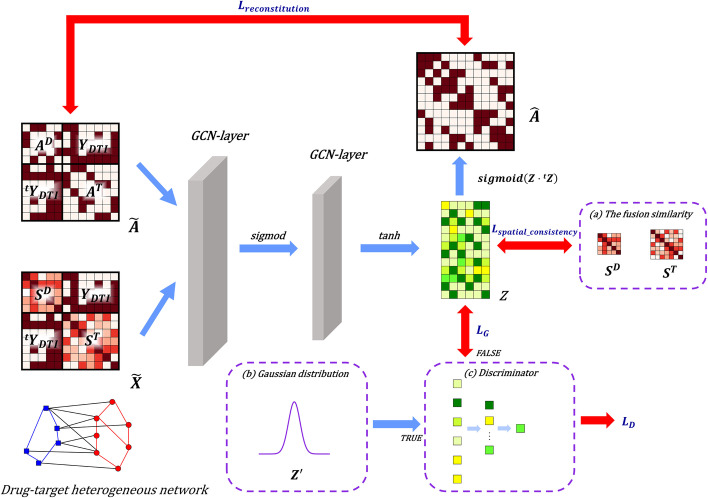
Encoder and decoder in SDGAE. **a** The fusion similarity in "Multiple similarities fusion" Section; **b** Gaussian distribution; **c** MLP-based discriminator

In order to learn the low-dimensional feature vectors of drugs and targets. An autoencoder based on GCN was used to encode hidden representations of nodes. The encoding and decoding processes are illustrated in Fig. 3.

Briefly, in order to contain the node$'$s own feature in the process of aggregating information, it was necessary to add a self-loop to the adjacency matrix, which was represented as $A^{\prime }=\widetilde{A}+I$, where *I* represents an $m+n$ dimensional identity matrix. Then, $A^{\prime }$ was normalised to obtain the Laplace matrix according to the following equation:

5 $$\begin{aligned} \bar{A}=\widetilde{D}^{-\frac{1}{2}} A^{\prime } \widetilde{D}^{-\frac{1}{2}} \end{aligned}$$

where $\widetilde{D}(i, i)=\sum _j A^{\prime }(i, j)$. SDGAE was designed with two graph convolutional layers. To obtain a *k*-dimensional feature vector, the encoding process could be described as follows:

6 $$\begin{aligned} Z=\phi _2\left( \bar{A} \phi _1\left( \bar{A} \widetilde{X} W_1\right) W_2\right) \end{aligned}$$

where $W_1 \in R^{(m+n) \times l}$ and $W_2 \in R^{l \times k}$ represents the weight matrices of the first and second GCN layers that can be trained. *l* denotes the dimension of the feature vector for each node in the hidden layer. $\phi _1$ and $\phi _2$ are the non-linear activation functions. In particular, in our model, $\phi _1(t)={\text {sigmoid}}(t)=\frac{1}{1+e^{-t}}$, $\phi _2(t)={\text {tanh}}(t)=\frac{e^{t}-e^{-t}}{e^{t}+e^{-t}}$. After two convolutional layers, the $Z \in R^{(m+n) \times k}$ matrix was obtained. The first *m* and last *n* rows of this matrix represent the feature vectors of the drugs and the targets, respectively.

### Decoder and reconstitution loss

The main purpose of the decoder was to reconstruct the topological adjacency matrix $\widetilde{A}$ of the heterogeneous network based on matrix *Z*. The reconstructed matrix $\hat{A}$ was calculated using the following equation:

7 $$\begin{aligned} \hat{A}(i, j)=\phi _1\left( z_i \cdot { }^t z_j\right) \end{aligned}$$

where $\hat{A}(i, j)$ represents the propensity of node *i* and node *j* to interact. Larger values indicated that the decoder predicted that the two nodes were more likely to interact with each other. $z_i$ and $z_j$ represent the low-dimensional feature vectors of the node *i* and node *j*, taken from the *i*-th and *j*-th rows of *Z*, respectively. ${ }^t z_j$ denotes the transposition of $z_j$. To make the reconstructed matrix $\hat{A}$ as consistent with $\widetilde{A}$ as possible, we used the Mean Squared Error loss function as follows:

8 $$\begin{aligned} L_{reconstitution}=\Vert \widetilde{A}-\hat{A}\Vert ^2=\sum _i \sum _j(\widetilde{A}(i, j)-\hat{A}(i, j))^2 \end{aligned}$$

### Spatial consistency constraint (SCC)

There may be many potential interactions between drugs or targets, however, not all of them have been discovered so far. As a result, $\widetilde{A}$ may suffer from serious label missing. If only the matrix $\widetilde{A}$ was used as the guidance signal to learn the low-dimensional feature vectors of drugs and targets, the nearest neighbour relationships between nodes may shift in the embedding space. Changes in these relationships may have a negative impact on DTI prediction. The main purpose of "Spatial consistency constraint (SCC)" Section was to reduce the affect of noise in $\widetilde{A}$ and keep the topology of the nodes unchanged. Based on the assumption that nodes close to each other in the original space should also be close to each other in the embedding space, SDGAE designed the following strategy.

#### Sparsification of the similarity matrices

The SCC in the model mainly constrained the *p*-nearest neighbours of the nodes. Specifically, for nodes that were *p*-nearest neighbours in the original space, their distances in the embedding space should be as small as possible. A *p*-nearest neighbour graph was generated based on $S^{D}$ and $S^{T}$ for the drugs and targets, respectively. Taking drug as an example, a *p*-nearest neighbour graph *N* could be obtained from the following equation:

9 $$\begin{aligned} \begin{aligned} N(i,j) = \left\{ {\begin{array}{*{20}l} {1,} &{} {j \in \mathcal {N}_{p} (i)} &{} {i \in \mathcal {N}_{p} (j)} \\ {0,} &{} {j \notin \mathcal {N}_{p} (i)} &{} {i \notin \mathcal {N}_{p} (j)} \\ {0.5,} &{} {{\text {otherwise }}} &{} {} \\ \end{array} } \right. \end{aligned} \end{aligned}$$

where $\mathcal {N}_p(i)$ was the set of *p*-nearest neighbours of the drug $d_i$. Drug $d_i$ itself was included in the *p*-nearest neighbours set, which could be either known drugs or unknown drugs. The *N* matrix could then be used to sparse $S^{D}$ in an operation that is represented as follows:

10 $$\begin{aligned} \hat{S}^D(i,j)=N(i,j) \cdot S^D(i,j) \end{aligned}$$

Therefore, for all the drugs, we obtained a sparse similarity matrix $\hat{S}^D \in R^{m\times m}$. The same procedure was performed for the target similarity matrix $S^{T}$, for which we obtained $\hat{S}^T \in R^{n\times n}$.

#### Constraint

The *Z* output from the graph convolutional autoencoder hold the feature vectors of the drugs and targets. The matrix consisting of the first *m* rows of *Z* is denoted by $Z^{D} \in R^{m\times k}$, where each row of $Z^{D}$ represents the feature vector of a drug. Similarly, the matrix consisting of the last *n* rows of *Z* is denoted by $Z^{T} \in R^{n\times k}$, where each row of $Z^{T}$ represents the feature vector of a target. Spatial consistency loss was defined as follows:

11 $$\begin{aligned} \begin{aligned} L_{spatial\_consistency}&=\lambda _l\left( \left\| Z^{D}\right\| _F^2+\left\| Z^{T}\right\| _F^2\right) \\&\quad +\lambda _d \sum _{i, r=1}^m \hat{S}^D(i,r)\left\| Z_{i}^{D}-Z_{r}^{D}\right\| ^2 \\&\quad + \lambda _t \sum _{j, q=1}^n \hat{S}^{T}(j,q)\left\| Z_{j}^{T}-Z_{q}^{T}\right\| ^2 \end{aligned} \end{aligned}$$

where $\lambda _l$, $\lambda _d$ and $\lambda _t$ were non-negative hyperparameters that controlled the weights of the three parts of the loss. $Z_{i}^{D}$ and $Z_{j}^{T}$ were the *i*-th and *j*-th rows of $Z^{D}$ and $Z^{T}$ respectively. The first term in Eq. (11) was the Tikhonov regularisation. Moreover, the second term measured the distance of the embeddings among drugs that were the nearest neighbours in the original space. The purpose of minimizing the second term was to ensure that drugs that were close to each other in the original space were also close to each other in the embedding space. With this term, it was guaranteed that the topology of the drug nodes remained essentially unchanged during representation learning. Similarly, the third term ensured that the topology of the target nodes also remained unchanged. Eq. (11) could be rewritten as:

12 $$\begin{aligned} \begin{aligned} L_{spatial\_consistency}=&\lambda _l\left( \left\| Z^D\right\| _F^2+\left\| Z^{T}\right\| _F^2\right) \\&\quad +\lambda _d {\text {Tr}}\left( { }^t Z^D \mathcal {L}^D Z^D\right) \\&\quad +\lambda _t {\text {Tr}}\left( { }^t Z^T \mathcal {L}^T Z^{T}\right) \end{aligned} \end{aligned}$$

where ${\text {Tr}}(\cdot )$ denotes the trace of a matrix. $\mathcal {L}^D=D^D-\hat{S}^D$ and $\mathcal {L}^T=D^T-\hat{S}^T$, respectively. Additionally, $D^D(i, i)=\sum _r \hat{S}^D(i, r)$ and $D^T(j, j)=\sum _q \hat{S}^T(j, q)$ were diagonal matrices. ${ }^t Z^D$ and ${ }^t Z^T$ were the transpose of $Z^D$ and $Z^T$ respectively.

By integrating $''$Decoder and reconstitution loss$''$ and "Spatial consistency constraint (SCC)" Section together with Eqs. (8) and (12), the loss of the encoder was obtained as follows:

13 $$\begin{aligned} L_{encoding}=L_{reconstitution}+L_{spatial\_consistency} \end{aligned}$$

### Adversarial model

To improve the robustness of the model and reduce noise interference in $\widetilde{A}$, a GAN model was designed. The purpose of GAN was to make the feature vectors more consistent with Gaussian distribution. A multilayer perceptron (MLP) was constructed to act as the discriminator *D*. In SDGAE, graph convolutional encoder also acted as the generator *G*. The loss functions of both the generator and discriminator were binary cross-entropy loss functions, which were defined as follows:

14 $$\begin{aligned} {\text {BCELoss}}(p, y)=-[y \log p+(1-y) \log (1-p)] \end{aligned}$$

where *p* represents the predicted output of the model and *y* denotes the sample label. As described in the "Graph convolutional encoder" Section, the feature vector matrix $Z \in R^{(m+n)\times k}$ of drugs and targets was obtained, with $z_i$ as the *i*-th row in *Z*. The matrix sampled from the true Gaussian distribution was $Z^{\prime } \in R^{(m+n)\times k}$, with $z_i^{\prime }$ as the *i*-th row in $Z^{\prime }$. The loss functions of the discriminator and the generator were as follows:

15 $$\begin{aligned} L_D= & {} \frac{1}{m+n} \sum _i{\text {BCELoss}}\left( D\left( z_i\right) , 0\right) +\frac{1}{m+n} \sum _i {\text {BCELoss}}\left( D\left( z_i^{\prime }\right) , 1\right) \end{aligned}$$

16 $$\begin{aligned} L_G= & {} \frac{1}{m+n} \sum _i {\text {BCELoss}}\left( D\left( z_i\right) , 1\right) \end{aligned}$$

To sum up, as $L_{encoding}$, $L_D$, and $L_G$ were optimised using the Adam algorithm [34], informative and robust feature vector matrix $Z \in R^{(m+n)\times k}$ of drugs and targets could be obtained. *Z* was subsequently used to predict the likelihood of DTIs.

### Classifier based on LightGBM

Due to the serious problem of class imbalance, ensemble learning has been used to alleviate its negative effects. Herein, LightGBM, which can efficiently address the class imbalance problem, was used as DTI prediction classifier in SDGAE. LightGBM can fully utilise the information of all negative samples.

In the representation learning stage, we obtained the feature vector matrix *Z* for the drugs and targets. The first *m* and last *n* rows of *Z* represent the feature vectors of the drugs and targets, respectively. If we used $Z(d_i)$ and $Z(t_j)$ to represent the feature vectors of the drug $d_i$ and target $t_j$, then the feature vector of the drug-target pair $(d_i,t_j)$ would be defined as a concatenation of $Z(d_i)$ and $Z(t_j)$; that is, $x(d_i, t_j)=Z(d_i) \oplus Z(t_j)$. The label of the sample $(d_i,t_j)$ was obtained from the matrix *Y*; that is $y(d_i, t_j)=Y(i, j)$. Therefore, we had a total of 1923 positive samples and 1,068,573 negative samples. The loss function of the classifier was binary cross-entropy loss function as follows:

17 $$\begin{aligned} L_{lightgbm}=\frac{1}{m \times n} \sum _i \sum _j {\text {BCELoss}}(\hat{Y}(i, j), Y(i, j)) \end{aligned}$$

where $\hat{Y}(i,j)$ was the classifier output of the sample $(d_i,t_j)$. By optimising the above-described loss, we obtained the interaction propensities among all drugs and targets ($\hat{Y}\in R^{m \times n}$). The higher the score of the LightGBM model output, the more likely it was that the drug-target pair could interact.

## Results

### Evaluation metrics

We used a 10-fold cross-validation approach [35] to evaluate the performance of the SDGAE model. Moreover, the receiver operating characteristic (ROC) curve [36] was constructed. The area under the ROC curve (AUC) [37] was used to assess the predictive performance of the model. However, as the number of negative samples in the dataset was significantly higher than that of the positive samples, in this case, the area under the precision-recall curve (AUPR) [38] could provide more information for assessing the overall performance of the model. Of note, AUC considers both positive and negative sample classification performance, whereas AUPR mainly focuses on positive samples and is suitable for highly unbalanced datasets [39]. Therefore, the AUC and AUPR are usually adequate metrics for evaluating the performance of a model for DTI prediction [40]. Many similar studies have used these two metrics to evaluate the performance of methods for predicting DTIs [26, 28, 41–43]. As biologists often select drug-target pairs with high prediction scores for subsequent wet experiment validation, the recall rates of the top $\omega$ (5%, 10%, 15%, 20%, and 30%) proportion of candidate targets predicted by the model were selected. The average recall rate for all drugs represented the ability of the model to recognise positive samples.

### Comparison with other methods

#### Compared methods and parameters setting

To further evaluate the performance of SDGAE, we compared it with several other state-of-the-art methods, including GRMF [8], DTINet [9], GANDTI [28], NGDTP [7], MolTrans [19], and GADTI [26]. The hyperparameters of these methods were selected based on ranges recommended in the literature. We set $\lambda _l=0.2$, $\lambda _d=0.1$, $\lambda _t=0.1$ in GRMF. The restart probability of the random walk in DTINet was set to $r=0.8$, as well as $k_1=100$, $k_2=400$. For GANDTI, we set $l=500$, $k=200$ and $a=2220$. For NGDTP, in the matrix factorisation stage, we set $a_1=a_2=a_3=0.1$, $f_r=280$ and $f_p=210$, whereas on the GBDT model, we set $num_{leaves}=80$ and $learning\ rate=0.02$. For MolTrans, we set $learning\ rate =0.0001$, $epoch=30$, $batch \ size=16$, and $dropout=0.1$. For GADTI, we set $learning\ rate=0.001$ and $d=1000$.

The programming language we used was Python (3.7). SDGAE was built using the GPU version of Pytorch (1.10.0). The main libraries used were lightgbm (3.3.3), torch_geometric (2.1.0), and sklearn (1.0.2). SDGAE was trained and optimised on NVIDIA GeForce RTX 3060. Lastly, the hyperparameters of the SDGAE were set as follows: $\eta =0.8$, $K=10$, $p=5$, $\lambda _l=1e\text {-}5$, $\lambda _d=0.001$, $\lambda _t=0.001$, $epoch=5000$, the $learning\ rate$ of the representation learning stage was 0.0001, and the $learning\ rate$ of the LightGBM model was 0.02.

#### Experimental comparison

**Fig. 4:**
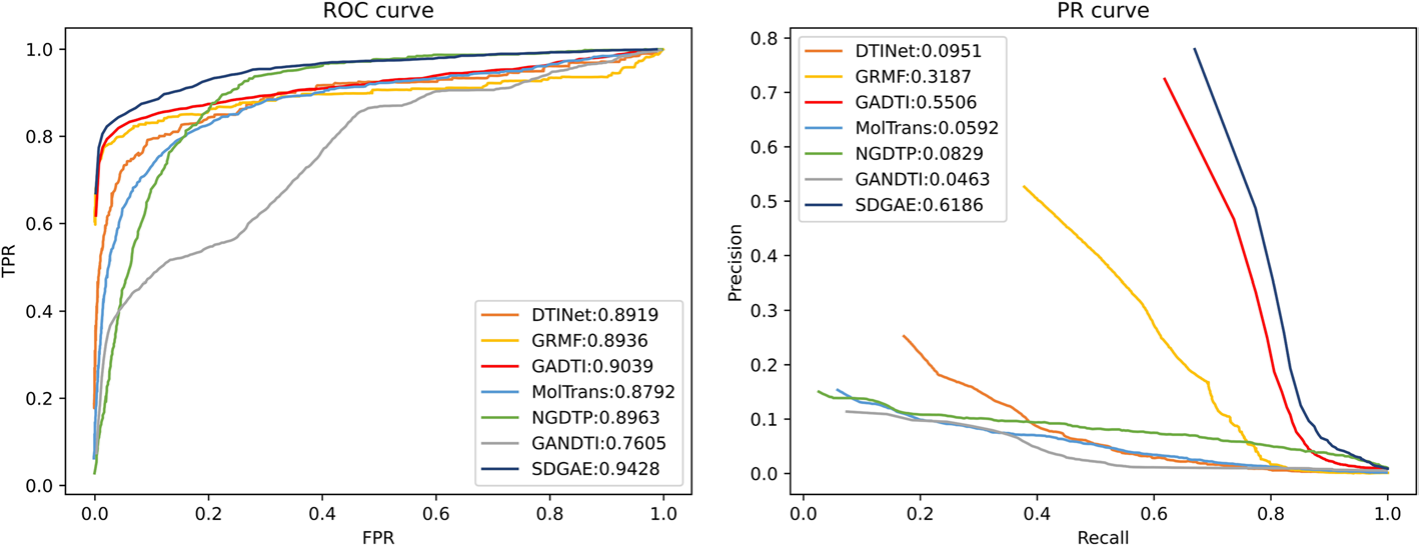
ROC and PR curves of SDGAE and other methods

The ROC and PR curves of each method are presented in Fig. 4. The AUC and AUPR are listed in Table 2. SDGAE achieved the best performance among the seven methods, with the AUC 3.89% higher than the second best model (GADTI) and the AUPR 6.80% higher than the second best model (GADTI). The AUC and AUPR of GRMF were 4.92% and 29.99% lower than those of SDGAE. In addition, the AUC and AUPR of DTINet were 5.09% and 52.35% lower than those of SDGAE. Furthermore, the AUC and AUPR of NGDTP were 4.65% and 53.57% lower than those of SDGAE respectively. Finally, the AUC and AUPR of MolTrans were 6.36% and 55.94% lower than those of SDGAE. GANDTI performed the worst among all seven methods, which may be due to the large number of unknown drugs and unknown targets in the dataset (159 unknown drugs and 1088 unknown targets). GANDTI was unable to effectively encode the features of isolated nodes, which limited its performance.

To demonstrate that the AUC and AUPR of SDGAE were higher than the other six methods from a statistical point of view, a *t*-test was implemented. For the predicted scores of each drug, we separately calculated the AUC and AUPR. AUC list and AUPR list of each method were obtained. The *P*-values between SDGAE and each compared method were calculated by *t*-test. The results are shown in Table 3. The results showed that SDGAE was significantly better than the other six methods at the significance level of 0.05 in terms of AUC and AUPR.

**Table 2:** AUC and AUPR of SDGAE and other methods

	DTINet	GRMF	GADTI	MolTrans	NGDTP	GANDTI	SDGAE
AUC	0.8919	0.8936	0.9039	0.8792	0.8963	0.7605	**0.9428**
AUPR	0.0951	0.3187	0.5506	0.0592	0.0829	0.0463	**0.6186**

The best results are highlighted with bold font

**Table 3:** Statistical results of SDGAE and other methods

	DTINet	GRMF	GADTI	MolTrans	NGDTP	GANDTI
*P*-value (AUC)	0.0229	0.0285	2.74e−4	0.0367	4.09e−3	1.13e−53
*P*-value (AUPR)	2.18e−23	9.90e−3	3.42e−13	6.83e-25	7.67e−93	9.86e−130

The *P*-values were calculated based on the AUCs and AUPRs of each drug

**Fig. 5:**
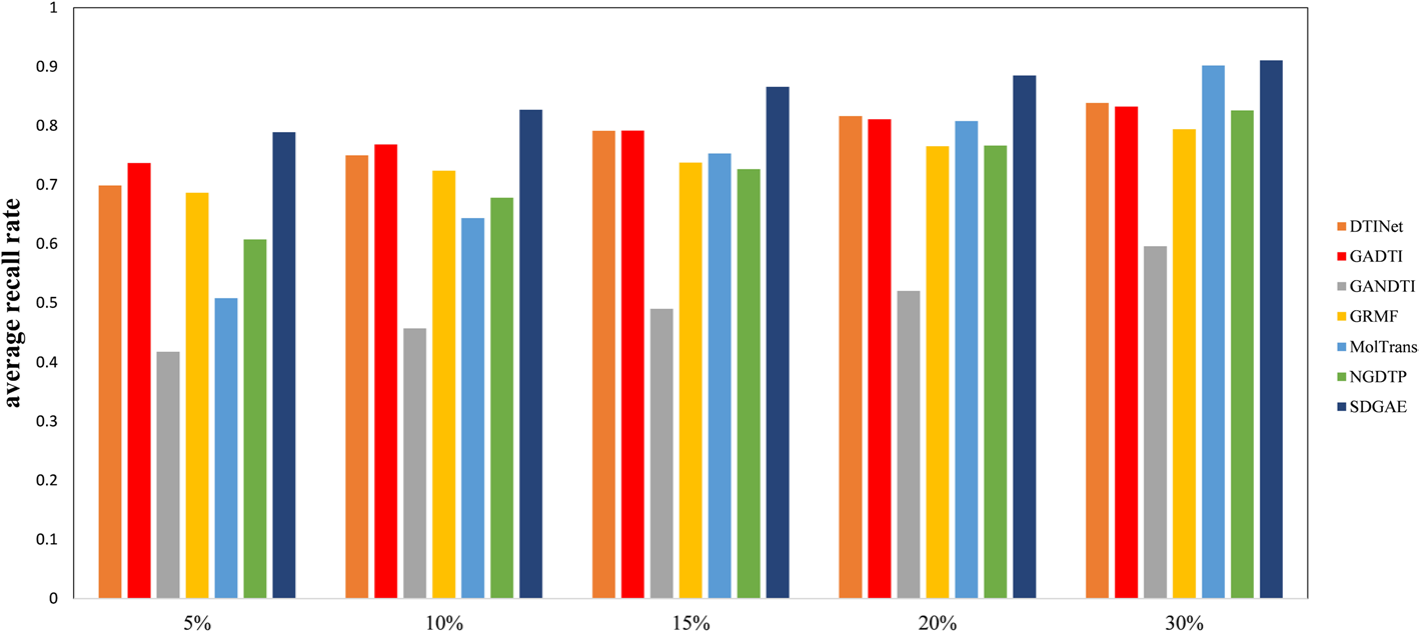
Average recall rates at different top $\omega$ cutoffs

Drug-target pairs with higher prediction scores will be further validated by biologists through wet-lab experiments. Thus for each drug, the recall rates of the top $\omega$ (5%, 10%, 15%, 20%, and 30%) candidate targets were collected as an indication of the ability of the model to identify DTIs. The higher the average recall, the more real DTIs are identified. Figure 5 illustrates that SDGAE had the highest average recall rate among the seven methods regardless of the $\omega$ selected, achieving average recall rates between 78.92% and 91.10%. When $\omega$ was 5%, 10%, 15%, 20%, and 30%, the average recall rates of SDGAE were higher than those of the second best method by 5.21% (GADTI), 5.87% (GADTI), 7.39% (GADTI), 6.87% (DTINet), and 0.90% (MolTrans), respectively. If $\omega$ was set to 5%, 10%, or 15%, then GRMF performed better than NGDTP. In turn, NGDTP performance was better than that of GRMF when $\omega$ was set to 30%. When $\omega$ was set to 20%, the performance of GRMF and NGDTP were similar.

**Fig. 6:**
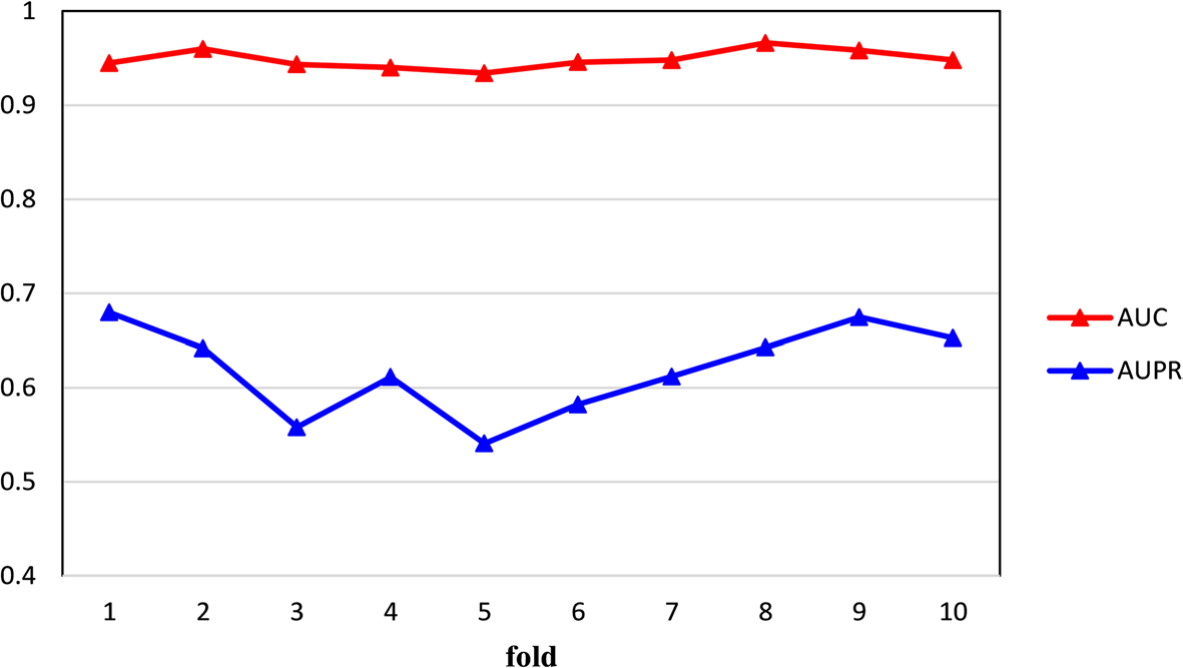
AUC and AUPR of each fold

Figure 6 illustrates the AUC and AUPR of each fold in the whole prediction process of SDGAE. From this figure, we can find that the AUC and AUPR of SDGAE were consistently high in each fold. In addition, the AUC and AUPR of each fold did not fluctuate much. Therefore, SDGAE has good robustness to DTI dataset.

### Ablation experiments

**Fig. 7:**
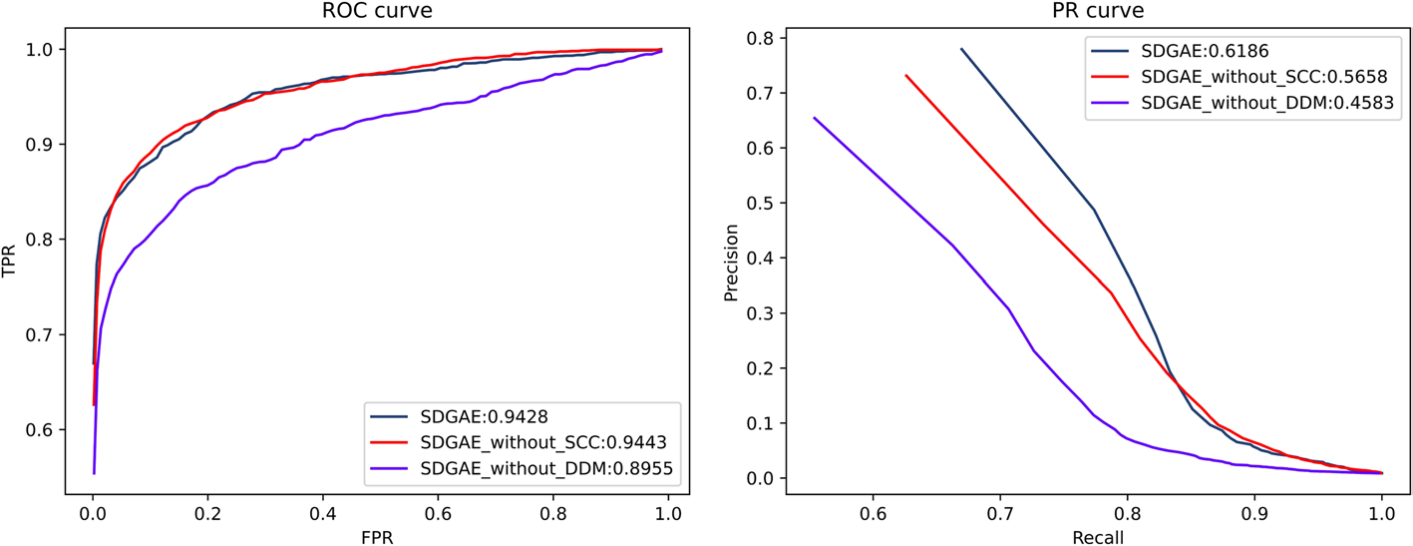
ROC and PR curves of each method

**Table 4:** Comparison of AUC and AUPR values for ablation experiments

	SDGAE	SDGAE_without_DDM	SDGAE_without_SCC
AUC	0.9428	0.8955	**0.9443**
AUPR	**0.6186**	0.4583	0.5658

The best results are highlighted with bold font

Next, the SDGAE model was further tested but without DDM (See "Densify DTI matrix (DDM)" Section), as well as without SCC (See "Spatial consistency constraint (SCC)" Section).

From Table 4 and Fig. 7, we can see that without DDM, the AUC and AUPR of the SDGAE model were 89.55% and 45.83%, respectively, which represented a significant reduction of 4.73% and 16.03%, compared with the original model. When SCC was not used, a slight AUC increase was observed (up by 0.15%), which was very small, whereas the AUPR of the model decreased significantly (down by 5.28%). Because there is a serious problem of class imbalance, AUPR is more important than AUC. Hence, if SCC was excluded, the performance of SDGAE also deteriorated significantly. Based on the results of the ablation experiments, we confirmed that both the DDM and SCC resulted in a significant improvement in the performance of the method.

If only $\widetilde{A}$ was used as the guidance signal to learn the low-dimensional feature vectors of drugs and targets (See "Graph convolutional encoder" Section), the nearest neighbour relationships between nodes in the embedding space could shift. Take drug as an example.

**Fig. 8:**
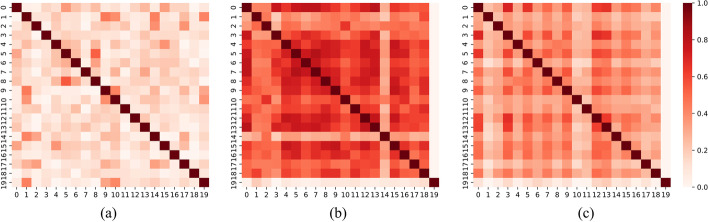
Relationships between 20 drugs in **a** original space, **b** embedding space without SCC and **c** embedding space with SCC, respectively. The deeper the colour, the more similar the two drugs are

Twenty drugs were randomly selected to observe the differences in feature vectors learned with and without the SCC. As shown in Fig. 8, the subplot (a) illustrates the similarity between 20 drugs sampled from $S^D$ matrix. This similarity was determined manually and we defined this space as original space. The subplot (b) is the similarity matrix between the feature vectors of 20 drugs learned without SCC. Correspondingly, the subplot (c) is the similarity matrix between the feature vectors of 20 drugs learned with SCC. It was observed that, if SCC was not used, the similarity between the feature vectors was much greater than that in the original space. The high similarity between feature vectors was not beneficial for subsequent DTI prediction. In contrast, if SCC was used, the similarity between the feature vectors were closer to the original space. Therefore, SCC really played a role in maintaining the graph structure. The nearest relationships between nodes in the embedding space remained as close as possible to the original space. This made the feature vectors more beneficial for subsequent DTI prediction.

### Predicting novel DTIs

To demonstrate the ability of SDGAE to discover potential DTIs, we used all known DTIs in the dataset and performed 10-fold cross-validation on negative samples to obtain the interaction propensity of all drug-target pairs in the dataset. In Table 5, we presented the 20 drug-target pairs with the highest scores predicted by the SDGAE. To verify the results of the model, we searched several public databases, including DrugBank [44], PubChem [45], DrugCentral [46], STITCH [47], and KEGG [48], for evidence of these 20 drug-target pair interactions.

**Table 5:** Top 20 of candidate drug-target pairs

Rank	Drug ID	Drug name	Protein ID	Protein name	Supported evidence
1	DB01215	Estazolam	P48169	GABRA4	PubChem
2	DB00829	Diazepam	P48169	GABRA4	KEGG
3	DB06800	Methylnaltrexone	P41143	OPRD1	STITCH
4	DB00335	Atenolol	P07550	ADRB2	DrugBank
5	DB00363	Clozapine	P21918	DRD5	KEGG
6	DB01019	Bethanechol	P11229	CHRM1	DrugBank
7	DB06216	Asenapine	P28221	HTR1D	KEGG
8	DB00734	Risperidone	P21918	DRD5	DrugCentral
9	DB00809	Tropicamide	P08912	CHRM5	STITCH
10	DB00734	Risperidone	P28222	HTR1B	KEGG
11	DB00799	Tazarotene	P48443	RXRG	KEGG
12	DB06216	Asenapine	P21918	DRD5	DrugCentral
13	DB00543	Amoxapine	P28223	HTR2A	DrugBank
14	DB01186	Pergolide	P34969	HTR7	STITCH
15	DB00411	Carbachol	P20309	CHRM3	DrugBank
16	DB00806	Pentoxifylline	Q08499	PDE4D	Literature [49]
17	DB01019	Bethanechol	P20309	CHRM3	DrugBank
18	DB00185	Cevimeline	P08172	CHRM2	KEGG
19	DB00454	Meperidine	Q8TCU5	GRIN3A	KEGG
20	DB00734	Risperidone	P34969	HTR7	DrugBank

Among the 20 drug-target pairs most likely to interact predicted by SDGAE, 7 were supported by KEGG database, 6 by DrugBank database, 3 by STITCH database, 2 by DrugCentral database and 1 by PubChem database. For the one remaining drug-target pair, we also found literature that indicates the interaction can occur, as noted by $''$Literature$''$ in Table 5. For all 20 drug-target pairs predicted by SDGAE, we can find evidence of existing interactions outside the dataset, demonstrating the powerful ability of SDGAE to predict potential DTIs. Refer to Additional file 1 for novel DTIs of all drugs predicted by SDGAE.

## Discussion

The results showed that both AUC and AUPR of SDGAE were higher than the other compared methods (Table 2, Fig. 4). AUPR, in particular, was substantially higher than other methods. We conjecture that the reason why SDGAE performs better than these methods is that it integrates the advantages and mitigates the disadvantages of these methods. Among these methods, DTINet leverages multiple association information and NGDTP can fully utilise negative samples information to effectively alleviate the class imbalance problem; however, both are shallow models with limited learning capabilities. GADTI and GANDTI are deep learning methods based on graph convolutional encoding, but GCNs do not perform well in networks with isolated nodes or sparse networks. In addition, GADTI and GANDTI do not consider the invariance of the nearest neighbour relationships between nodes during representation learning. In comparison, SDGAE is a method based on graph convolutional autoencoder and it has a powerful learning capability. SDGAE measures similarity from multiple perspectives, which makes full use of information from multiple data sources. Moreover, the LightGBM in SDGAE makes full use of the information from negative samples and alleviates class imbalance problem by building multiple decision trees. SDGAE densifies adjacency matrix to deal with isolated nodes in heterogeneous networks, fully exploiting the effectiveness of GCN. In addition, SCC operation maintains the nearest neighbour relationships between nodes unchanged, which is beneficial for the subsequent training of the classifier. As an outcome of its enhanced efficacy, SDGAE identified more potential DTIs than the other methods, which paves the way for a faster discovery of potential drug targets. Ablation experiments showed that both the SCC and DDM significantly improved the performance of the model. Finally, all 20 novel DTIs predicted by SDGAE were supported by several published works, which demonstrates the powerful ability of SDGAE for DTI prediction.

Compared with the work of others, we paid more attention to the changes occurring in the nearest neighbour relationships of the nodes in the process of representation learning (Fig. 8). Without SCC, nodes that were not close to each other in the original space would likely become close to each other in the embedding space after representation learning. We believe that an important reason for this is that $\widetilde{A}$ contains noise. There are some interactions that are not yet discovered. SDGAE was designed to reduce the interference of these false labels. From Fig. 8 and Table 4, it could be concluded that intentionally keeping the nearest neighbours unchanged during representation learning is beneficial for DTI prediction to some extent.

Although SDGAE was only used to predict missing DTIs in this work, SDGAE is a versatile method. If the similarity between nodes is defined, SDGAE can be easily applied to other link prediction problems, such as the predictions of microRNA-small molecule [50–53], drug-side effect [54, 55], gene-disease [56–58], and microRNA-disease [59, 60] associations. In the future we will investigate the performance of the SDGAE in other link prediction problems. In addition, the coronavirus disease 2019 (COVID-19) has become a major global health problem [61] and is still haunting the entire human race. However, researching and designing a new drug for patients with COVID-19 may take a lot of time. Drug repurposing may be an effective alternative [62]. We will apply SDGAE model to the datasets which contain more targets and drugs related to COVID-19. In other words, SDGAE will be used to predict potential therapeutic drugs for the treatment of COVID-19 in the future [62, 63].

## Conclusions

We propose a novel method, SDGAE, for DTI prediction. During the representation learning stage, the idea of maintaining graph structure was used to make the topology of nodes in the embedding space closer to the original space. Thus, the nearest neighbour relationships between nodes in the embedding space remained as close as possible to the original space. In order to alleviate the disadvantage that GCN cannot encode isolated nodes, the DTI matrix was first densified to reduce the number of isolated nodes in heterogeneous networks. This operation fully exploited the effectiveness of the GCN.

Taken together, this study provides a good inspiration for DTI prediction models based on graph neural network encoding. The idea of SCC and DDM can be applied to other methods without difficulty. Thus, it provides a general idea for the optimisation of DTI prediction methods based on graph neural network encoding.

## Supplementary Information

**Additional file 1**. Novel DTIs predicted by SDGAE.xlsx: it contains 30 candidate targets for all drugs in the dataset. The candidate targets for each drug are sorted in descending order according to their prediction scores.

## Data Availability

The source code is available at https://github.com/936773184/SDGAE. The dataset used in these experiments is available online at https://github.com/luoyunan/DTINet.

## Abbreviations

DTI: Drug-target interaction
SCC: Spatial consistency constraint
DDM: Densify DTI matrix
GCN: Graph convolutional network
ROC: Receiver operating characteristic
GAN: Generative adversarial network
RWR: Random walk with restart
DDI: Drug-drug interaction
TTI: Target-target interaction
MLP: Multilayer perceptron.
